# Associations Between Nutritional Status, Frailty and Health-Related Quality of Life Among Older Long-Term Care Residents in Helsinki

**DOI:** 10.1007/s12603-019-1320-9

**Published:** 2020-01-27

**Authors:** K. S. Salminen, M. H. Suominen, H. Kautiainen, K. H. Pitkälä

**Affiliations:** 1 Department of General Practice and Primary Health Care, University of Helsinki, Finland, POB 20, FIN-00014 Helsinki, Finland; 2 Vantaa Social Welfare and Health Care, Vantaa, Finland; 3 Helsinki University Hospital, Unit of Primary Health Care, Helsinki, Finland

**Keywords:** MNA, nutrition, frailty, health-related quality of life, long-term care

## Abstract

**Objectives:**

The aim of this study was to examine how nutritional status modifies the association between frailty and health-related quality of life (HRQoL) among older nursing home residents. We also investigated how residents’ energy intake is linked to frailty score.

**Design and participants:**

A total of 486 older (> 65 years of age) nursing home residents living in Helsinki, Finland were included to this cross-sectional study.

**Methods:**

We collected data on the residents’ background information, HRQoL by 15D, nutritional status by Mini Nutritional Assessment (MNA), frailty status (Fried’s phenotype criteria; pre-frail: 1–2 criteria and frail: 3–5) and energy intake (one- or two-day food records).

**Results:**

The frail residents were more often malnourished and had lower HRQoL than those in the prefrail group. Energy and protein intakes were significantly lower among frail women than prefrail women. Energy intake was linearly associated with frailty points. When residents in the frail and prefrail groups were divided according to their nutritional status, both nutritional status and frailty were associated with HRQoL, but there was no interaction.

**Conclusions:**

Both nutritional status and frailty were associated with HRQoL, and lower energy intake indicated a higher frailty score. An adequate energy intake may promote residents’ HRQoL and prevent frailty in long-term care.

## Introduction

Malnutrition is common among nursing home residents. According to the Mini Nutritional Assessment (MNA), its prevalence has varied from 14% to 21% and its risk from 45% to 53% in Europe (1). In Zwienen-Pot et al.’s (2018) study only 18% of nursing home residents had adequate energy and protein intake (2). Malnutrition has been associated with lower health-related quality of life (HRQoL) among community dwelling and institutionalized older people (3–6). The predictors of malnutrition among nursing home residents have been cognitive impairment, depression, functional impairment, and swallowing difficulties (7).

Frailty is associated with poor nutritional status, and good nutrition is an important mean to prevent frailty (8, 9). A meta-analysis found that 12 out of 15 studies of community-living elderly people associated malnutrition with frailty (8). Malnutrition and frailty share common risk factors such as weight loss and functional impairment (8). Nutrition intake is associated with several frailty criteria, including low muscle strength, feelings of exhaustion, reduced physical activity and slow walking speed (10). Frailty, in turn, can reduce nutrition intake and have negative consequences for nutritional status (10).

In addition to nutritional status, previous studies have found low energy intake to be associated with frailty among community-living older people (11, 12). Although studies investigating the associations between frailty and energy intake among nursing home residents are scarce, it is known that energy intake is often inadequate in nursing homes (13–15). In their study, Jyväkorpi et al. found that even older residents with good nutritional status had inadequate energy intake (13).

The prevalence of frailty in nursing homes has ranged from 19% to 75.6% (16). Frailty has no simple definition, and its prevalence varies depending on the method used to evaluate it (17). Although it has been associated with reduced HRQoL in institutional settings (18–20), less is known about which factors modify the association between frailty and HRQoL. It is important to identify these factors and develop ways to promote residents’ HRQoL in nursing homes. As nutritional status affects frailty criteria, it has the potential to modify the association between frailty and HRQoL.

To our knowledge, no previous studies have explored the interplay between nutritional status, frailty and HRQoL among older nursing home residents. The aim of this study was to investigate how nutritional status modifies the association between frailty and HRQoL among older long-term care residents. We also investigated how energy intake is associated with frailty score.

## Methods

Participants were recruited from three nursing homes and 14 assisted living facilities in Helsinki. These assisted living facilities are like nursing homes but have a more home-like environment. These facilities also include group homes for patients with dementia. Assistance is available around the clock and a registered nurse is in charge of the ward.

We recruited the first 549 volunteer residents in these settings. The inclusion criteria for the present study were: age ≥ 65 years, living permanently in institutional care, sufficient information available on demographic factors and frailty status according to Fried’s phenotype criteria, 1- or 2-day food record, and HRQoL according to 15D instrument. A total of 486 met these criteria and were included in the study.

The local ethics committee of Helsinki University Hospital approved the study. We acquired informed consent from all participants or in cases of moderate-severe dementia (Mini-Mental State Examination (MMSE < 20 points), from their closest proxies.

In each ward, trained nurses collected the data. The information on the residents’ demographic information, diagnoses and current use of medications were retrieved from medical records. The residents’ dependency in activities of daily living (ADL) was assessed with the Clinical Dementia Rating Scale (CDR) “personal care” question (1=totally independent; 2=needs prompting, 3=requires assistance in dressing, personal hygiene, and keeping of personal belongings, 4=requires much help with personal care; often incontinent) (21). CDR “personal care” ≥; 2 was defined as dependence in ADLs. Cognitive status was measured using the Mini Mental State Examination (MMSE) (22).

The residents were divided into two groups according to their clinical stage of frailty as determined by the modified Fried criteria (23). In this study, the five slightly modified frailty criteria were: 1) Unintentional weight loss > 5% during the preceding three years (yes/no). 2) Exhaustion - based on residents’ or nurses’ reported low energy most or all of the time during the preceding four weeks. 3) Low physical activity - the question inquired whether the residents exercised regularly weekly (yes/no) - “no” was taken to denote low physical activity. 4) Slowness - based on the walking speed in the Short Physical Performance Battery test (SPPB) (< 0.85 m/s). 5) Physical weakness - based on self-reported difficulty (not at all=0) carrying or lifting a grocery bag. Residents who met one or two of the above criteria were classified into the prefrail groups, and residents meeting three or more criteria were classified into the frailty groups. Only two residents met none or one criterion, and these were classified into the prefrail group.

We used the MNA to evaluate nutritional status. The MNA gives a maximum score of 30 points. Less than 17 points indicates malnutrition, 17–23.5 a risk of malnutrition, and 24 or more good nutrition status (24, 25). In addition, each resident was weighed and their body mass index (BMI) was calculated as weight divided by height squared (kg/m^2^).

Residents’ energy and protein intakes were determined by a 1- or 2-day food record kept by ward nurses. We analyzed the food records using AivoDiet dietary software (version 2.2.0.0, Aivo Oy, Turku, Finland), which contains the Fineli Food Composition database Release 16 (2013). This database includes recipes for the typical Finnish mixed dishes that are usually served in long-term care. The instruction was to record all the foods and beverages consumed by the resident. The nurses estimated portion sizes using household measures. For prepacked products, the exact brand and product name was required.

Nutrition care was assessed by asking the ward nurses several questions such as whether the participant ate normal (normal or soft) food or liquid/pureed food (yes/no). The amount of the offered food eaten by the resident was elicited by asking “How much does the residents eat of the main meal on average?” with five response options: “eats only a little, eats less than half, eats half their meal, eats most of their meal, or eats all or nearly all of their meal”. The responses “eats only a little” and “eats less than half” were dichotomized as eats less than half, and the response “eats half their meal, “eats most of their meal” or “eats all or nearly all of their meal” as eats more than half. The portion was compared to model images of food portions.

The 15D instrument, a validated, generic tool for measuring HRQoL, was used to evaluate the residents’ HRQoL (26). Its dimensions are mobility, vision, hearing, breathing, sleeping, eating, speech, excretion/elimination, usual activities, mental function, discomfort and symptoms, depression, distress, vitality, and sexual activity. The 15D can be completed during a conversation with the resident but also by proxy who knows the patient well. It combines the advantages of a 15-dimention profile and a single-index measure (26). A score of 0 indicates the poorest HRQoL and 1 indicates perfect HRQoL. The 15D index is reliable, sensitive and responsive to change (26). The nurse most familiar with the resident was interviewed for the 15D if the subject was unable to respond due to poor cognition.

Statistical comparisons between the groups were made using the t-test or chi-square test. The relationship between frail points and standardized energy intake per day was evaluated using the analysis of covariance (ANCOVA) with an appropriate contrast (linearity). Similarly, the relationship between MNA and score for frailty was analyzed. Age and gender were used as covariates in these models. The normality of variables was evaluated graphically and by the Shapiro-WilkW test. The Stata 16.0 (StataCorp LP; College Station, Texas, TX, USA) statistical package was used for the analysis.

## Results

The study population consisted of 486 older, long-term care residents. Of these, 79.6% were female and 69.5% were frail. The study population characteristics of the frail group are provided in Table 1. The residents in the frail group were more often dependent in ADL functions, had a lower MMSE score, had a lower BMI and were more often malnourished than the residents in the prefrail group. The female residents in the frail group had lower energy and protein intake than the female residents in the prefrail group. Among the male residents, energy or protein intake did not differ significantly in the groups. The residents in the frail group ate pureed or liquid food more often than the residents in the prefrail group. The mean 15D index was 0.69 (SD 0.11) among the residents in the prefrail group and 0.57 (SD 0.12) among the residents in the frail group. The difference was significant (p < 0.001).

**Table 1:** Population characteristics according to frailty status

**Population characteristics**	**Prefrail N = 148**	**Frail N = 338**	**p-value**
*Backgroud characteristics*			
Age, mean (SD*)	83(8)	83(8)	0.57
Female (%)	119(80)	268(79)	0.78
Education (≤ 8 years), n (%)	63(48)	133(44)	0.45
Dependence in ADLs †, n (%)	120(83)	320(96)	&lt; 0.001
MMSE ‡, mean (SD)	14.4(6.4)	12.2(7.2)	0.003
*Nutrition status*			
BMI §, mean (SD)	27.4(4.6)	25.2(5.1)	&lt; 0.001
MNAll, n (%)			&lt; 0.001
Malnourished (&lt; 17 points)	7(6)	57(20)	
At risk of malnutrition (17–23.5 points)	83(66)	195(68)	
Normal nutritional status (> 23.5 points)	35(28)	33(12)	
*Nutrition intake*			
Energy kcal, mean (SD)			
Women	1686(394)	1539(385)	&lt; 0.001
Men	1781(330)	1821(378)	0.63
Protein, total g, mean (SD)			
Women	58.6(16.8)	51.8(14.9)	&lt; 0.001
Men	62.6(12.7)	63.7(16.0)	0.76
Protein g/kg, mean (SD)	0.84(0.26)	0.84(0.28)	0.82
*Nutrition care*			
Eats normal food, n (%)	129(87)	187(55)	&lt; 0.001
Eats less than half of the food portion, n (%)	24(16)	72(22)	0.17
*HRQOL*			
15D score **, mean (SD)	0.69(0.11)	0.57(0.12)	&lt; 0.001

* SD=Standard deviation; † ADL= Activities of daily living measured by CDR=Clinical Dementia Rating Scale ‘personal care’ score ≥ 2 (Hughes et al. 1982) (21); ‡ MMSE= Mini-Mental State Examination (Folstein et al. 1975) (22); § BMI=body mass index (kg/m2); ll MNA=Mini Nutritional Assessment (Guigoz 2006) (25); **(Sintonen 2001) (26)

**Figure 1:**
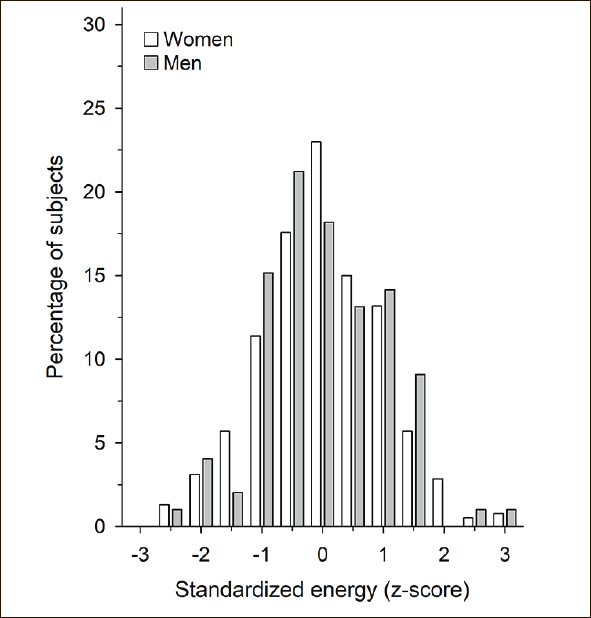
Gender-specific standardized (z-score) energy intake/day. Energy intakes were standardized by z-scores to include both men and women in the same analyses

**Figure 2:**
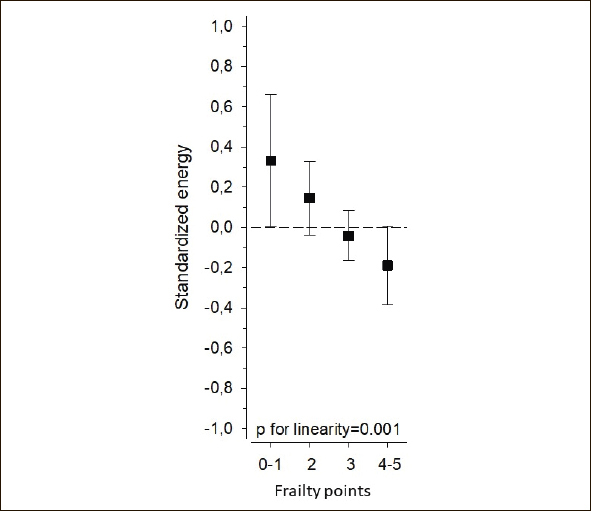
Relationship between frailty points (Fried et al.2001)(23) and gender-specific standardized energy intake per day (p=0.001). Adjusted for age

Figure 1 shows the gender-specific standardized energy intake per day (Z scores). There was a linear association between the frailty score according to Fried’s criteria and gender-specific standardized energy intake (p = 0.001) (Figure 2). The higher gender-specific standardized energy intake was associated with lower frailty points.

**Figure 3:**
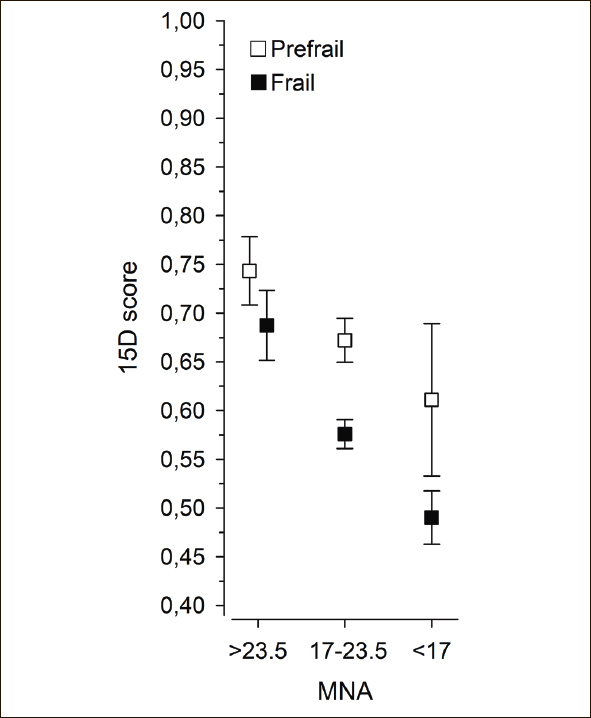
Relationship between Mini Nutritional Assessment (MNA) (Vellas et al. 1999)(24) and health-related quality of life (HRQoL) (Sintonen 2001)(26) in prefrail and frail groups. Both the frailty group (p < 0.001) and the MNA score (p < 0.001) were linearly associated with HRQoL, but the interaction was not significant (p = 0.18). Adjusted for age and gender

Figure 3 shows the relationship between MNA score and HRQoL in the prefrail and frail groups. Both the frailty group (p < 0.001) and the MNA score (p < 0.001) were linearly associated with HRQoL, but the interaction was not significant (p = 0.18).

## Discussion

In this study, the frail and prefrail residents in institutional settings differed in many ways: the frail residents were more dependent and malnourished and had lower cognition and lower HRQoL. The frail women also showed lower protein and energy intakes than the prefrail women. The frailty points were linearly associated with gender-specific standardized energy intake. Furthermore, both nutritional status according to MNA and frailty according to Fried’s phenotype criteria were significantly associated with HRQoL among older institutionalized residents. However, there was no interaction.

The strength of this study is the large sample size of nursing home residents and the fact that detailed food records and frailty assessments were available. We assessed frailty on the basis of Fried’s criteria, which is widely used (27). We measured the residents’ HRQoL using the 15D tool, which can be completed by proxy and can thus also be used for residents with severe cognitive impairment (26). A limitation of this study was its cross-sectional nature, which meant that we could not determine the causality between frailty, nutritional status and HRQoL. To our knowledge, this is the first study to investigate the interaction between HRQoL, nutritional status and frailty in an institutional setting.

The prevalence of frailty in this study was 70%, which is quite high compared with prior studies (16). Our sample also included residents with severe dementia. It is not surprising that the prefrail and frail residents differed in respect to their functioning, nutritional status and BMI. Previous studies have associated both nutritional status and higher dependency in ADL functions with frailty (8, 10), and frailty with lower HRQoL (18–20). In Buckinx et al.’s study of older nursing home residents, all HRQoL dimensions were significantly poorer among the frail residents, except bodily pain (18). However, Buckinx et al.’s study did not include residents with cognitive impairments. In their study, Serrano et al. found the physical and mental components score of SF-12 to be significantly poorer among frail residents than among robust residents (20).

Logically, the frail women’s energy and protein intakes were lower than those of the prefrail women. This was not the case among the men. However, the number of men in both the frail and prefrail groups was relatively low.

In our study, the gender-specific standardized energy intake was linearly associated with the frailty score. Previous studies have often estimated energy and nutrition intake using a food frequency questionnaire (FFQ) or 24h recall, rather than food records as we used in our study (10, 11, 28). As in our study population, most of the residents had moderate or severe cognitive impairments, FFQ and 24h recall would not have been suitable methods to evaluate their food intake (29). In Bartali et al.’s study of community living residents, energy intake of under 21 kcal/kg was associated with an increased risk of frailty (11). In a Rotterdam follow-up study, every 100 kcal-increase in energy intake decreased the odds of frailty by 5% (28). In the same study, most of the participants lived independently. As weight loss is one frailty components and reduced energy intake is closely associated with it, it is not surprising that standardized energy intake was associated with frailty points. The link between frailty and nutrition may be body composition (10). Decreased energy intake may lead to insufficient protein and micronutrient intake, which in turn can lead to wasting of muscle mass (10). Low energy intake can lead to feelings of exhaustion and thus reduce physical activity (11, 30).

In our study, nutritional stage according to MNA was linearly associated with HRQoL. Previous studies among non-institutionalized older people and nursing home residents have associated poor nutrition status with poor HRQoL (3–6). The MNA and 15D both include questions on ADL functions, so associations between the MNA and HRQoL are not unexpected. In institutional settings, better nutritional status has been associated with less dependency in ADL functions (31–33).

The frail residents in our study had poorer HRQoL than the prefrail group residents at all nutritional stages of MNA. It seems that the stages of frailty and malnutrition have a linear relationship with HRQoL, but that this association has no interaction. It may be questioned whether the MNA measures items similar to frailty, as it contains items such as BMI, dementia, mobility, reduced food intake, and weight loss, which may overlap with items defining frailty.

## Conclusions

This study reveals that both nutritional status and frailty are associated with HRQoL among older nursing home residents. It also shows that energy intake is linearly associated with frailty points. Adequate nutrition intake may have the potential to prevent frailty in long-term care settings and to maintain HRQoL.

## Notes

*Ethical standards:* The local ethics committee of Helsinki University Hospital and the City of Helsinki approved the study. This study was supported by the Päivikki and Sakari Sohlberg Foundation. The funder played no role in the study design, analysis or interpretation of data, nor in writing the report or the decision to submit this article. The authors were independent researchers not associated with the funder.
